# Swelling Property and Metal Adsorption of Dialdehyde Crosslinked Poly Aspartate/Alginate Gel Beads

**DOI:** 10.3390/polym18020177

**Published:** 2026-01-08

**Authors:** Takuma Yamashita, Toshihisa Tanaka

**Affiliations:** 1 Graduate School of Medicine, Science and Technology, Shinshu University, 3-15-1 Tokida, Ueda 386-8567, Nagano, Japan; 22hs110h@shinshu-u.ac.jp; 2 Faculty of Textile Science and Technology, Shinshu University, 3-15-1 Tokida, Ueda 386-8567, Nagano, Japan

**Keywords:** poly aspartic acid, alginic acid, dialdehyde crosslinked, hydrogel bead, swelling, metal adsorption

## Abstract

Dialdehyde crosslinked poly aspartate/alginate hydrogel beads were synthesized by covalently introducing poly aspartate into the alginate network via dialdehyde-mediated crosslinking, and the resulting effects on swelling and adsorption behavior were investigated. Alginate was partially oxidized to form dialdehyde alginate and crosslinked with poly aspartic acid via Schiff base formation, followed by ionic crosslinking with calcium ions. The chemical structure and morphology of the gel beads were characterized by Fourier transform infrared spectroscopy and scanning electron microscopy. Incorporation of PAsp significantly altered the swelling behavior of alginate-based gel beads. In saline solution, PAsp-modified gel beads exhibited a swelling ratio of approximately 112 g/g, which was higher than that of calcium alginate gel beads. This behavior is suggested to be associated with changes in the alginate–calcium network structure induced by polymer modification. PAsp-modified gel beads exhibited moderate but distinct adsorption behavior depending on the adsorbate. Removal efficiencies of approximately 40–50% were observed for copper and cobalt ions, while a removal efficiency of around 50% was obtained for the cationic dye crystal violet. In contrast, adsorption of the anionic dye Congo red decreased with increasing PAsp content, indicating charge-dependent adsorption behavior. Overall, this study demonstrates that PAsp modification via dialdehyde-mediated crosslinking influences both the swelling and adsorption properties of alginate-based hydrogel beads. The results provide fundamental insight into how network modification can be used to tune the behavior of alginate-based hydrogels in aqueous environments.

## 1. Introduction

In recent years, hydrogels have been used in a variety of applications such as hygiene products, biomedical applications, wastewater treatment, and soil conditioners. Hydrogels are polymeric materials with three-dimensional networks of crosslinked, water-soluble polymers. Some hydrogels are used as water-absorbent polymers and can absorb 100 to 1000 times their own weight in water [1].

Currently, the mainly water-absorbing polymers are petroleum-derived and non-biodegradable polymers such as sodium polyacrylate and polyacrylamide. These materials present several environmental concerns, including the depletion of resources, energy consumption and generation of toxic gases during incineration, landfill disposal issue, and environmental leakage. Therefore, water-absorbent polymers with rapid environmental degradation and low toxicity are increasingly required [2,3].

On the other hand, some water-absorbing polymers show biodegradable properties. Representative examples include poly aspartic acid and alginic acid. Poly aspartic acid is a polymer obtained by polymerizing aspartic acid, an amino acid naturally occurring in biological systems. It is an acidic poly amino acid that possesses additional free carboxylic acid residue in addition to those involved in the peptide bond. These carboxylic acid residues can bind monovalent cations such as sodium ions, resulting in increased water absorbency similar to that of polyacrylic acid [4,5]. At present, aspartic acid is produced from petroleum-derived fumaric acid and ammonia; however, research on fermentation-based production methods is progressing [6]. In addition to its biodegradability, poly aspartic acid exhibits biocompatibility, low toxicity, and metal adsorption capability. Accordingly, it has been investigated for applications such as dye removal membranes and wound dressing materials [7,8]. In this study, we prepared sodium poly aspartate (PAsp), the sodium salt form of poly aspartic acid.

Alginic acid is a natural polysaccharide derived from brown algae and is composed of two monosaccharide units, *α*-*L*-guluronic acid and *β*-*D*-mannuronic acid [9]. Each monomer unit contains two hydroxyl groups and one carboxylic group, imparting hydrophilicity to the polymer. However, alginic acid itself is not soluble in water due to strong intermolecular hydrogen bonding. Water solubility is achieved when the carboxylic groups of alginic acid are converted to their monovalent cation salts, resulting in enhanced water absorbency [10]. Alginic acid is also well known to form hydrogel through ionic crosslinking with multivalent cations such as calcium [11]. The structure formed between molecular chains of *α*-*L*-guluronic acid blocks (G blocks) during gelation is referred to as an egg-box structure [12]. Alginate-based materials exhibit biocompatibility, metal adsorption properties, and non-toxicity, and have been extensively studied for biomedical, drug delivery, and water treatment applications [9,13,14]. Recent research has summarized the use of alginate-based hydrogels for water treatment applications. These studies particularly highlight their potential for heavy metal adsorption [15]. Furthermore, dialdehyde alginate (ADA), obtained by oxidation of alginate, exhibits excellent biodegradability and reactive aldehyde groups capable of forming Schiff base linkages with amine-containing polymers [16]. In this study, ADA was used as a crosslinking agent with PAsp [17].

The purpose of this study is to investigate how the incorporation of poly aspartic acid into calcium crosslinked alginate hydrogel beads via dialdehyde-mediated covalent bonding influences swelling behavior and adsorption tendencies under different aqueous conditions. Previous studies by Ali Pourjavadi et al. and Suo et al. reported enhanced swelling ratios of hydrogels achieved by grafting acrylic acid onto saccharide polymer chains [18,19]. However, these systems relied on toxic chemical crosslinkers for acrylic acid grafting. In contrast, PAsp was selected in this study as an alternative to acrylic acid because it contains free carboxylic groups similar to polyacrylic acid while remaining biodegradable and biocompatible. Polymer composite gels of poly aspartic acid and alginate have been investigated for drug delivery and dye adsorption applications [11,20]. Nevertheless, the combined effect of PAsp incorporation via dialdehyde crosslinking and subsequent calcium ion crosslinking on swelling and adsorption behavior, particularly under saline conditions, have not been systematically clarified. In addition, other studies have reported the crosslinking of gelatin and dialdehyde alginate to fabricate artificial blood vessels and edible films [17,21]. However, investigations focusing on comparative swelling behavior in different aqueous environments together with adsorption tendencies for metal ions and dyes remain limited. In this study, alginate and poly aspartic acid were crosslinked using ADA, and the swelling behavior of the resulting gel beads was evaluated in distilled water, saline solutions, and phosphate-buffered saline. The adsorption behavior toward metal ions and dyes was also investigated under fixed experimental conditions to clarify the influence of PAsp incorporation.

## 2. Materials and Methods

### 2.1. Materials

Sodium alginate (SA) (1000 cps.) was purchased from Nacalai Tesque Corporation (Kyoto, Japan). L-aspartic acid (Asp), polyglutamic acid (molecular weight = 300,000–500,000 (PGA)), L-lysine hydrochloride (Lys), phosphoric acid, diisobutyl ketone (DIBK), sodium periodate, ethylene glycol, ethanol, hydroxyl amine hydrochloride, methyl orange, anhydrous calcium chloride, sodium chloride, phosphate-buffered saline (without calcium and magnesium), concentrated hydrochloric acid, 0.1 mol/L sodium hydroxide solution, copper standard solution (1000 ppm dissolved in 0.1 M nitric acid), cobalt standard solution (1000 ppm dissolved in 0.1 M nitric acid), crystal violet, Congo red, and concentrated nitric acid were purchased from FUJIFILM Wako Pure Chemical Corporation (Osaka, Japan).

### 2.2. Synthesis of Sodium Poly Aspartate

Lysine with a free amino group was added to increase the branch of PAsp and the crosslinking point between PAsp and dialdehyde alginate, and PGA was added to extend the molecular chain. Asp/Lys/PGA 34.5 g/5 g/0.5 g, DIBK 80 g as a solvent and phosphoric acid 15 g as a catalyst were mixed in a 4-neck flask [22,23,24]. The flask was set up with a Dean–Stark apparatus, thermometer, and stirrer, and heated in an oil bath at 180 °C for 7 h to obtain polysuccinimide (PSI), a precursor of poly aspartic acid. After heating, DIBK and synthetic PSI were separated by filtration. After filtration, filter residue was washed by ethanol and distilled water for removing residual DIBK and phosphoric acid, and dried under reduced pressure. PSI was dissolved in 0.1 M sodium hydroxide at 50 °C to hydrolysis for obtaining PAsp [25]. PAsp solution was lyophilized by the freeze dryer (FD-100e, Tokyo Rikakikai Co., Ltd., Tokyo, Japan) to a powder and stored in a desiccator.

### 2.3. Preparation of Dialdehyde Alginate

ADA was synthesized according to the method of Soumya R.J. et al. Sodium alginate (SA) 10 g was added to ethanol 50 mL and dispersed with stirring [26]. Sodium periodate 2.7 g was dissolved in distilled water 50 mL, added to the SA dispersion, and reacted with stirring overnight under dark conditions at room temperature in order to obtain ADA. After stirring, the reaction of sodium periodate was stopped by adding ethylene glycol 10 mL, and the ADA dispersion was placed in a dialysis tube (molecular weight 3500 cut) and dialyzed multiple times with distilled water to remove the impurities. After dialysis, the ADA dispersion was lyophilized. The obtained samples were dissolved in distilled water to make a film and analyzed by Fourier transform infrared spectroscopy (FT-IR (FT/IR-6600, JASCO Corporation, Tokyo, Japan)), and the oxidation degree of ADA was determined by hydroxylamine titration method [21].

Hydroxylamine hydrochloride reacts with aldehyde groups to generate 1 mol of hydrogen chloride per 1 mol of aldehyde group. The indicator for the titration was prepared by using a 0.25 N hydroxylamine hydrochloride solution with distilled water and adding 1 drop of 0.05 wt% methyl orange solution [17,21]. The prepared ADA 0.1 g was added to hydroxylamine solution 20 mL and stirred for 2 h. Then, 0.1 mol/L sodium hydroxide solution was dropped by burette to measure the oxidation degree of ADA. For the blank, the same amount of SA solution was used. The oxidation degree of ADA was determined by the average value of oxidation degree (OD) from three titrations. The OD of the ADA solution was calculated by Equation (1) [27]:

(1) $$\mathrm{OD}\;(\%)\;=\;\frac{\left(V_{NaOH}-V_{blank}\right)\times N}{m\times M_{w\;\mathrm{alginate}\;repaeting\;\mathrm{unit}}}\;\times 100$$

where *V*_NaOH_ was the consumed volume (L) of sodium hydroxide by the ADA solution, *V*_blank_ was the consumed volume (L) of sodium hydroxide by the sodium alginate solution, *N* was the concentration of sodium hydroxide (sodium hydroxide concentration was 0.1 mol/L), *m* was the weight of ADA (g), and *M*_w_ was the molecular weight of the alginate repeating unit (198 g/mol).

### 2.4. Preparation of SA Composite Gel Beads

SA solution, SA-ADA solution, and SA-ADA-PAsp solution were prepared using distilled water as a solvent. SA solutions were prepared as SA = 2, 3 wt% in water (S2, S3), SA-ADA solutions were prepared by mixing SA = 1 wt% and ADA = 1 wt% in water (SD), SA-ADA-PAsp solutions were prepared by mixing SA/ADA/PAsp = 1/0.5/0.5, 1/1/1, 1/1/3, 1/1/5 (wt%/wt%/wt%) in water (SDP0.5, SDP1, SDP3, SDP5). All gel beads were prepared by dropping the mixed solution (20 mL) into 5 wt% calcium chloride aqueous solution (50 mL) using a syringe pump and immersing it overnight. The gel beads were isolated from the calcium chloride solution and washed several times with distilled water to remove residual calcium chloride solution. The gel beads were then lyophilized for 24 h and stored in vials.

### 2.5. Characterization of SA Composite Gel Beads

The internal structure of the fabricated gel beads was observed by scanning electron microscopy (SEM (JSM6010A, JEOL Co., Ltd., Tokyo, Japan)). Gel beads were cut open with a design knife and placed on a specimen stage covered with carbon tape, and platinum coating was performed (Ion sputter, JFC-1600, JEOL Co., Ltd., Tokyo, Japan). The cross section was then observed by SEM at a voltage of 10–15 kV. Attenuated Total Reflection (ATR) of FT-IR spectroscopy was used to analyze the chemical structure in the gel beads. 16 scans were performed in the range of 4000–400 cm^−1^ with a resolution of 4 cm^−1^ for FT-IR analysis.

### 2.6. Swelling Property

The swelling property of a single gel bead was investigated by weighing and immersing the bead in distilled water for 24 h. After immersion, the sample was wiped and weighed, and the swelling ratio was calculated from Equation (2) [28]. The swelling ratio in saline solution (0.9 wt% NaCl) and phosphate-buffered saline solution (PBS) were also measured using the same method.

(2) $$\mathrm{Swelling}\;\mathrm{ratio}\;[g/g]=\frac{W_{s}-W_{0}}{W_{0}}$$

*W*_0_ and *W_s_* were the initial weight and swelling weight of gel beads, respectively.

### 2.7. Metal Adsorption Property

Copper (Cu^2+^) and cobalt (Co^2+^) ions were selected as metal ions of adsorbent material. Metal adsorption was analyzed by ultraviolet–visible absorption spectroscopy (UV–vis (UV-2600/2700, Shimadzu Corporation, Kyoto, Japan)). Absorbance was measured in a single scan over a range of wavelength from 200 to 900 nm. Calibration curves were created from each of the obtained specific wavelengths using the metal ion solutions with concentrations at 0–1000 ppm [12]. Gel beads 0.01 g were immersed in each solution (10 mL, 50 ppm) for 24 h. After separating the gel beads, the concentration of metal ion solutions was measured by UV–vis spectroscopy, and the rate of decrease in metal ions concentration was calculated by using calibration curves. Removal rate of the metal ion was calculated with Equation (3). The metal ion contents in gel beads were analyzed by Energy Disperse X-ray Spectroscopy (EDS (JSM6010A, JEOL Co., Ltd., Tokyo, Japan)), 10 scans with voltage 10 kV, to compare the content of metal ions in gel beads before and after immersion [29].

(3) $$\mathrm{Removal}\;\mathrm{rate}\;(\%)=\frac{{(C}_{0}-C_{t})}{C_{0}}\;\times 100$$

*C*_0_ and *C_t_* were the initial concentration and after adsorption concentration of the metal ion solution, respectively.

### 2.8. Dye Adsorption Property

The difference in the dye adsorption property in the distilled water and saline solution was investigated by UV–vis spectroscopy. Crystal violet and Congo red were selected as dyes of adsorbent material. Calibration curves were created in the same protocol of the metal adsorption. To construct calibration curves, dye solutions were prepared by dissolving 1–20 mg/L of crystal violet and 5–30 mg/L of Congo red in distilled water and saline solution. Gel beads 0.01 g were immersed in the dye solution with a concentration of 10 mg/L (10 mL) for 24 h. After separating the gel beads, dye solutions were analyzed by UV–vis spectroscopy, and the rate of decrease in dye concentration was calculated by using calibration curves.

## 3. Results and Discussions

### 3.1. Synthesis of Sodium Poly Aspartate

**Figure 1:**
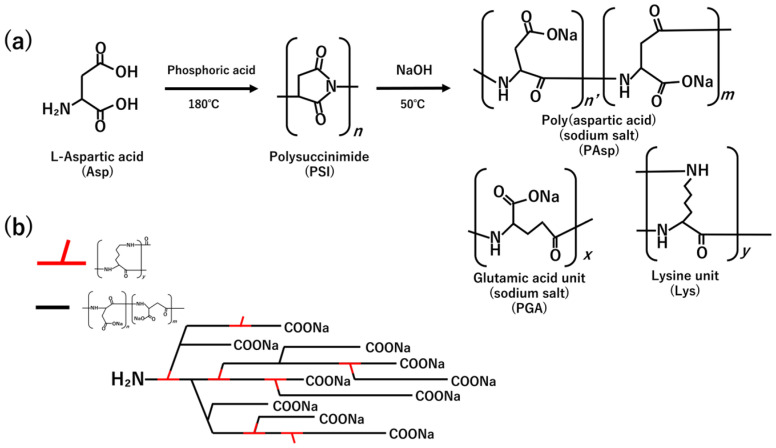
Images of (**a**) synthesis route of poly amino acid and (**b**) branched structure.

Sodium poly aspartate (PAsp) was synthesized as a precursor for preparing alginate–poly aspartic acid composite hydrogels. PAsp was prepared via thermal polycondensation of aspartic acid (with other amino acid as comonomers) to form polysuccinimide (PSI), followed by alkaline hydrolysis (Figure 1) [22,30].

**Figure 2:**
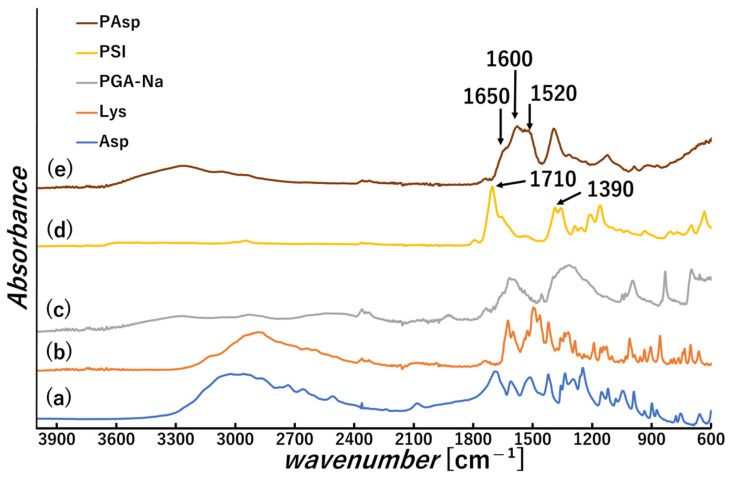
FT-IR spectra of (a) L-aspartic acid (Asp), (b) L-lysine (Lys), (c) polyglutamic acid (PGA), (d) polysuccinimide (PSI), and (e) poly aspartic acid sodium salt (PAsp).

PSI was successfully synthesized according to the procedure reported in the literature [22], and its chemical structure was confirmed by FT-IR (KBr method). In Figure 2(d), the absorption band at approximately 1710 cm^−1^ corresponds to the imide C=O stretching vibration, while the band near 1390 cm^−1^ is attributed to C-N stretching characteristic of imide groups [31].

PSI was then dispersed in 0.1 M sodium hydroxide solution (500 mL) and hydrolyzed at 50 °C under stirring to obtain PAsp. The resulting PAsp solution was lyophilized to obtain PAsp powder. The obtained sample was analyzed by FT-IR (Figure 2(e)), and C=O bond peak derived from the amide group (Amide I) and a N-H bond peak derived from the amide group (Amide II) appeared around 1650 cm^−1^ and 1520 cm^−1^, respectively, [32] and a peak derived from amino group around 3000–3600 cm^−1^ became larger compared to PSI (Figure 2(b)). This change suggests PSI was converted to PAsp [31].

Previous studies have reported two different structural models for poly (aspartic acid-co-lysine) prepared via thermal polycondensation followed by hydrolysis. Jiang et al. proposed that lysine predominantly reacts through its α-amino group during the formation of polysuccinimide (PSI), leaving the ε-amino group unreacted as a pendant side chain, resulting in a linear copolymer bearing pendant primary amines (pendant-type structure) [30]. This structural model assumes limited participation of ε-amino groups in condensation, producing water-soluble copolymers under moderate reaction conditions (160 °C, ~3.5 h).

In contrast, a patent on poly aspartic acid synthesis explicitly notes that excessive polycondensation temperatures can induce the formation of structurally complex motifs in the resulting polymer, which may adversely affect biodegradability, indicating that the reaction pathway becomes less selective at higher temperatures [22]. From a mechanical standpoint, elevated temperatures can promote side reactions and multi-point condensation during polysuccinimide formation, increasing the likelihood of branching or partial crosslinking even in poly aspartic acid homopolymers. When lysine is introduced into such systems, its bifunctional nature (α- and ε-amino groups) may further increase the probability of multi-point condensation, leading to statistically branched or partially crosslinked architectures rather than strictly linear pendant-type copolymers.

Given that the present synthesis was conducted at 180 °C for 7 h in the presence of phosphoric acid, which promotes imide formation and subsequent rearrangement, the copolymer is more likely to possess a partially branched structure rather than the idealized pendant-type linear structure. (Figure 1b) This structural tendency is further supported by the observed decrease in metal ion adsorption and salt-swelling behavior at higher poly aspartate content, suggesting restricted chain mobility and dense network formation, consistent with a branched structure.

### 3.2. Preparation of Dialdehyde Alginate

**Figure 3:**
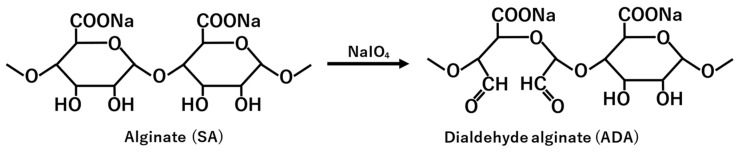
Synthesis route of dialdehyde alginate from sodium alginate.

Dialdehyde alginate (ADA) was prepared for crosslinking between alginate and poly aspartate. ADA is a substance formed by the cleavage of C2–C3 bond in the alginate monomer using sodium periodate and has two aldehyde groups in the monomer (Figure 3).

**Figure 4:**
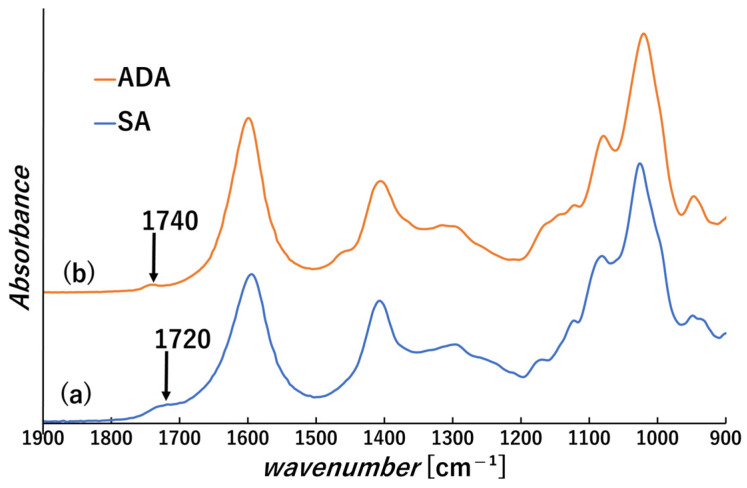
FT-IR spectra of (a) sodium alginate (SA) and (b) dialdehyde alginate (ADA).

Acidification of the solution accompanying the oxidation was observed, with the pH at the end of the oxidation reaction being approximately 4.8. This result suggests that the majority of the carboxylic groups in alginate still exist in the COO^−^ state [33]. From the FT-IR analysis of ADA (Figure 4(b)), the peak was identified around 1740 cm^−1^ and is considered to be a C=O bond derived from the aldehyde group [17]. However, a peak near 1720 cm^−1^ was also observed in the IR peak of SA. It is difficult to detect the aldehyde group of dialdehyde polysaccharide due to the formation of hemiacetals and hemialdals [34].

From the result of hydroxylamine titration, the aldehyde content of ADA was 31.2 ± 2.3% (*n* = 3) relative to the theoretical maximum, corresponding to an oxidation degree of approximately 15% per alginate monomer unit assuming dialdehyde formation. This result confirms successful introduction of aldehyde functionalities via periodate oxidation. The oxidation degree determined by hydroxylamine titration demonstrates that alginate dialdehyde with a controlled aldehyde content was successfully prepared for covalent crosslinking with PAsp.

### 3.3. Preparation of SA Composite Gel Beads

**Figure 5:**
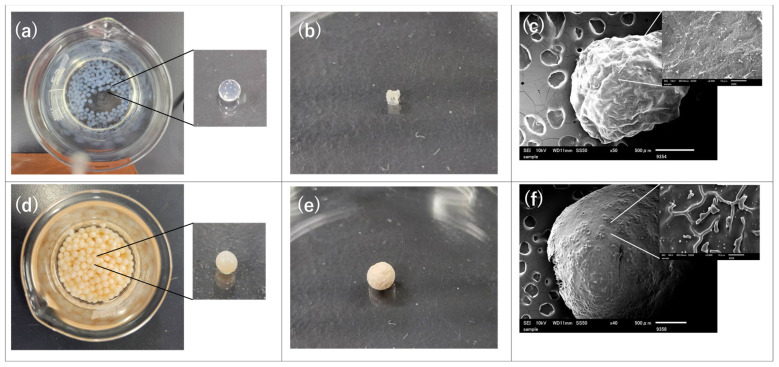
Changes in gel bead shapes and difference in surface morphology photographs of (**a**) prepared S2 gel bead, (**b**) dried S2 gel bead, (**d**) prepared SDP5 gel bead, and (**e**) dried SDP5 gel bead, and SEM images of (**c**) dried S2 gel bead and (**f**) dried SDP5 gel bead.

Several gel beads were prepared from solutions with different polymer ratios. The polymer ratios of the prepared gel beads are shown in Table 1. Gel beads were prepared by dropping the polymer solution using a syringe (10 mL) equipped with a 21-gauge needle and pumped at a flow rate of 0.6 mL/min into 50 mL of 5 wt% calcium chloride solution (Figure 5a,d). After washing several times with distilled water, the gel beads were lyophilized (Figure 5b,e). S2 and SDP5 exhibited different surface morphologies (Figure 5c,f). Characteristic surface wrinkles were observed on SDP5, whereas S2 showed a relatively smoother surface. According to Li et al., the incorporation of gelatin into alginate gel beads induced surface wrinkling, which was attributed to disruption of the internal homogeneity of the alginate gel matrix [35]. A similar surface feature was observed in the present PAsp-containing system. Such surface wrinkling is likely associated with structural heterogeneity and differential shrinkage occurring during the freezing and sublimation processes in lyophilization. The covalent introduction of PAsp may locally alter the hydrophilicity and the uniformity of the polymer network within the alginate matrix, leading to non-uniform stress distribution during solvent removal. As a result, the PAsp-containing beads tend to exhibit a wrinkled surface morphology after lyophilization, whereas the more homogeneous S2 beads retain a relatively smoother surface.

**Figure 6:**
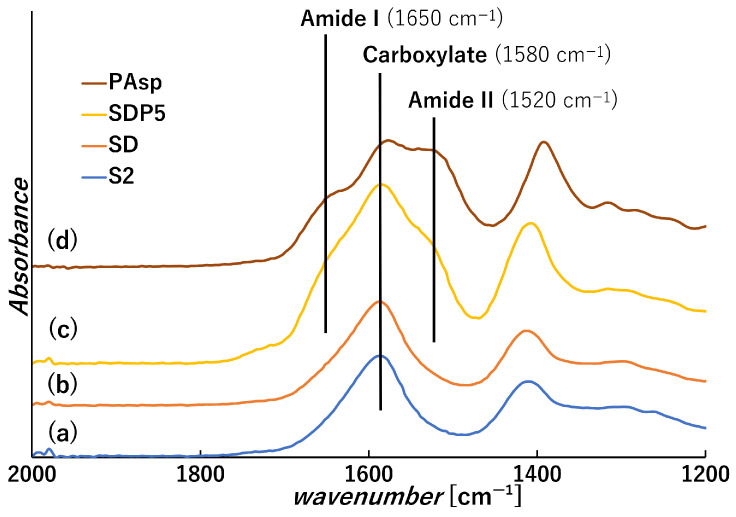
FT-IR spectra of (a) S2, (b) SD, (c) SDP5 dried gel beads, and (d) PAsp film.

**Figure 7:**
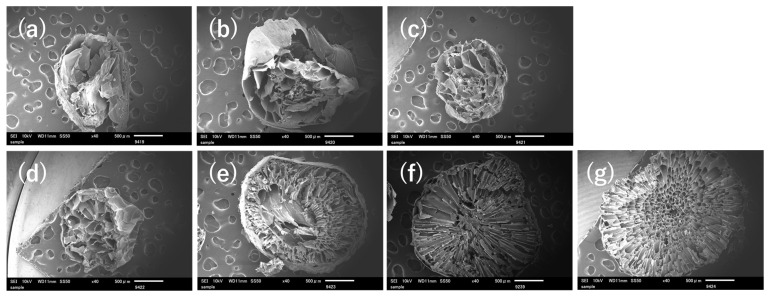
SEM images of section morphology of gel beads (**a**) S2, (**b**) S3, (**c**) SD, (**d**) SDP0.5, (**e**) SDP1, (**f**) SDP3, and (**g**) SDP5.

**Figure 8:**
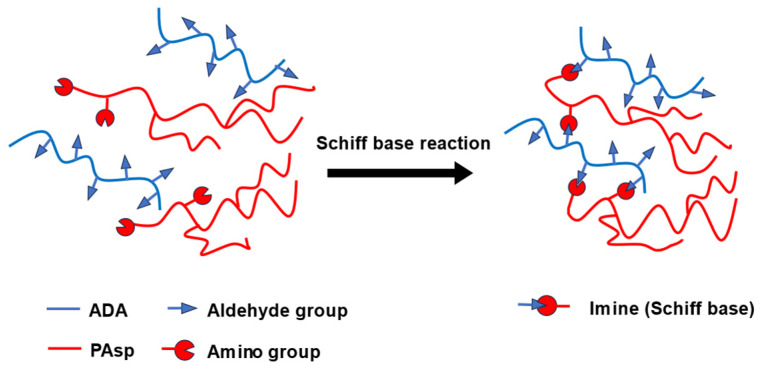
Schiff base formation between amino groups of PAsp and aldehyde groups of oxidized sodium alginate.

The obtained gel beads were analyzed by FT-IR and SEM, as shown in Figure 6 and Figure 7, respectively. FT-IR spectra showed that the introduction of PAsp resulted in the appearance of amide I (1650 cm^−1^) and amide II (1520 cm^−1^) bands, in addition to the characteristic peaks of S2 (Figure 6), indicating the presence of PAsp within the gel beads. This suggests that imine bonds may be formed between the aldehyde groups of ADA and the amino groups of PAsp via Schiff base reactions (Figure 8). Although a distinct imine peak around 1640 cm^−1^ was not clearly resolved, this may be due to overlap with other strong absorption bands in the same region [17]. In addition, the color of the SDP series solutions gradually changed to brown with increasing PAsp content. A similar color change has been reported for polypeptides reacted with aldehyde crosslinkers during Schiff base formation [17], supporting the occurrence of imine linkages in this system. SEM observation revealed that the inner morphologies of gel beads exhibited irregular, honeycomb-like appearances, which are commonly associated with the calcium crosslinked alginate network based on egg-box structures. In contrast, SDP1, 3, and 5 samples (Figure 7e–g) showed finer and more compact-looking internal features compared with S2 and SD; however, these features were spatially heterogeneous and did not form uniformly defined pores, suggesting a non-uniform internal network organization rather than a clearly defined porous structure. The SDP0.5 sample exhibited a morphology similar to that of SD, but different from that of S2, despite having the same polymer ratio. This difference may be related to variations in the internal network organization associated with PAsp incorporation, rather than polymer ratio alone [36,37]. These observations qualitatively confirm that chemically crosslinked PAsp/alginate gel beads with stable spherical morphology were successfully fabricated and were suitable as a structural basis for subsequent swelling and adsorption evaluations.

### 3.4. Swelling Property

#### 3.4.1. Swelling Property in Distilled Water

**Figure 9:**
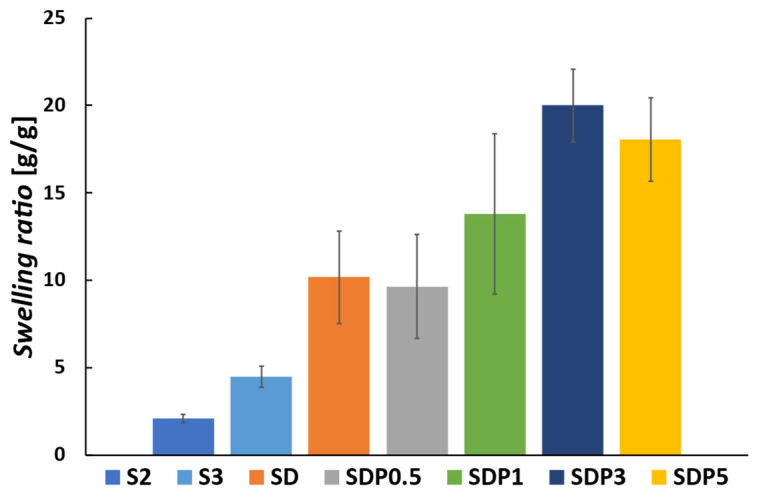
Swelling ratio of gel beads after immersion in distilled water [g/g]. The swelling ratio was measured after 24 h of immersion (*p*-values are summarized in Table S1).

The absorbing solution and swelling are important properties of hydrogels. Each dried gel bead was weighed, immersed in distilled water, and the swelling ratio was measured after 24 h. The results are shown in Figure 9, where the swelling of S2 and S3 were 2.1 g/g and 4.4 g/g, respectively. In the case of SD, the increase in water absorption may be related to oxidative cleavage of the alginate chains during ADA preparation, which can reduce the number of guluronic acid units and thereby decrease the number of Ca^2+^ crosslinking sites [38]. The increase in swelling ratio observed with increasing PAsp content may be associated with differences in the internal morphology of gel beads, as suggested by the SEM images in Figure 7. The presence of radially oriented structural features may facilitate water penetration into the beads during swelling. However, since a quantitative evaluation of porosity or pore size distribution was difficult, in this study, the discussion is limited to qualitative morphological observations.

#### 3.4.2. Swelling Property in Salt Solution

**Figure 10:**
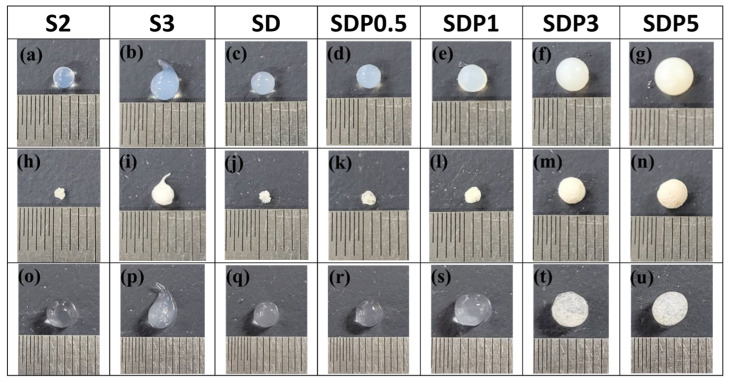
Photographs showing morphological changes in beads during preparation and swelling. (**a**–**g**) Gel beads before drying, (**h**–**n**) dried gel beads, and (**o**–**u**) gel beads after immersion in saline solution for 24 h.

**Figure 11:**
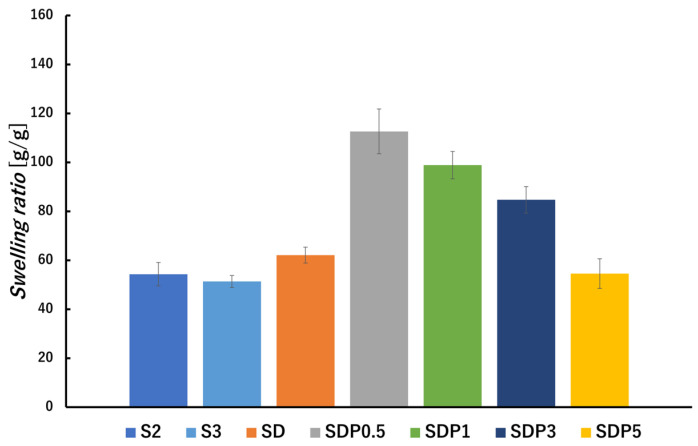
Swelling ratio of gel beads after immersion in saline solution [g/g]. The swelling ratio was measured after 24 h of immersion (*p*-values are summarized in Table S2).

Swelling measurements were also performed in a saline solution and PBS. After immersion in saline solution for 24 h, the swelling ratio was calculated. The images of gel beads at the time of gel preparation, after drying, and after immersion in saline solution are shown in Figure 10. It shows that the size of the beads changes during preparation (Figure 10a–g) and after immersion in saline solution (Figure 10o–u). The results showed that all gel beads exhibited higher swelling ratios in saline solution compared to distilled water (Figure 11). This behavior may be associated with change in ionic interaction within the Ca^2+^ crosslinked alginate network under saline condition, such as ion exchange between calcium ions in the gel and monovalent cations in the solution, which can influence the stability of the egg-box structures formed by G blocks [39].

In general, polyanionic hydrogels are known to exhibit reduced swelling in saline environments due to electrostatic charge screening [40,41]. Among the studied samples, SDP0.5 exhibited the highest swelling ratio in saline solution. While SD and SDP0.5 showed similar internal morphologies in SEM images (Figure 7), SDP0.5 exhibited a higher swelling ratio under saline conditions. This difference may be related to the presence of a limited amount of PAsp, which modifies polymer–water interactions under saline conditions. Conversely, further increasing the PAsp content resulted in a decrease in swelling ratio, suggesting that excessive PAsp incorporation may restrict network expansion in saline solution [4].

**Figure 12:**
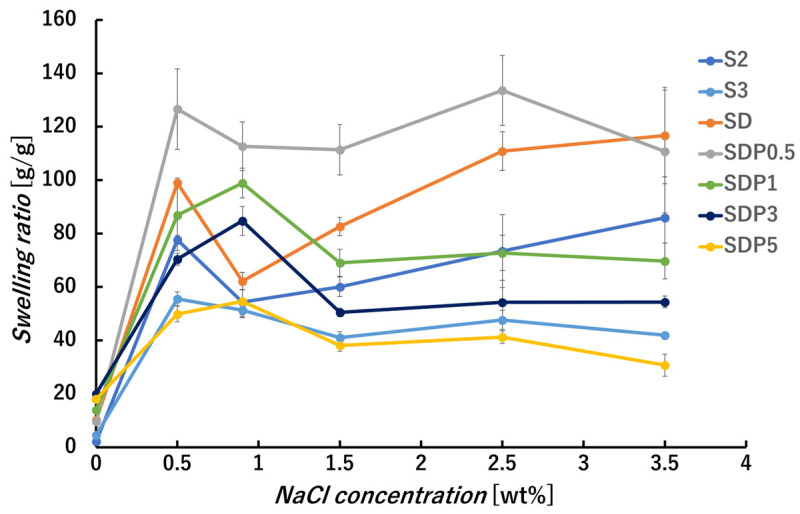
Swelling ratio of gel beads as function of NaCl concentrations [g/g]. The swelling ratio was measured after 24 h of immersion.

Swelling measurement was also performed at different NaCl concentrations (Figure 12). At low concentration (0–0.5%), the swelling ratios of all gel beads increased with increasing salt concentration. In the range of 0.5–0.9%, different trends were observed depending on the sample composition. While the swelling ratio of S2, S3, SD, and SDP0.5 gel beads decreased with increasing NaCl concentration. The SDP series exhibited slight increases within this range. At moderate NaCl concentration (0.9–1.5%), the swelling ratios of gel beads increased again, whereas those of S3 and the SDP series decreased. In contrast, SDP0.5 exhibited only a minor change in swelling ratio and remained nearly constant in this concentration range. At higher NaCl concentration (1.5–3.5%), the swelling ratios of S2 and SD further increased. SDP0.5 also showed an increase in swelling at intermediate concentrations, followed by a decrease at higher salt concentrations, which may be related to partial dissolution under these conditions. In contrast, S3 and the SDP series exhibited nearly constant swelling ratios over this concentration range.

These non-monotonic swelling behaviors suggest that multiple, competing effects govern the response of alginate-based gel beads under saline conditions. At lower salt concentrations, electrostatic screening can suppress polymer chain expansion, whereas at higher salt concentrations, ion exchange between Ca^2+^ and Na^+^ may influence the effective ionic interactions within the gel network. The distinct responses observed among S2, SD, SDP0.5, and the SDP series further indicate that PAsp incorporation modifies the balance between these effects, leading to composition-dependent swelling behavior rather than a uniform enhancement of swelling or dissolution.

**Figure 13:**
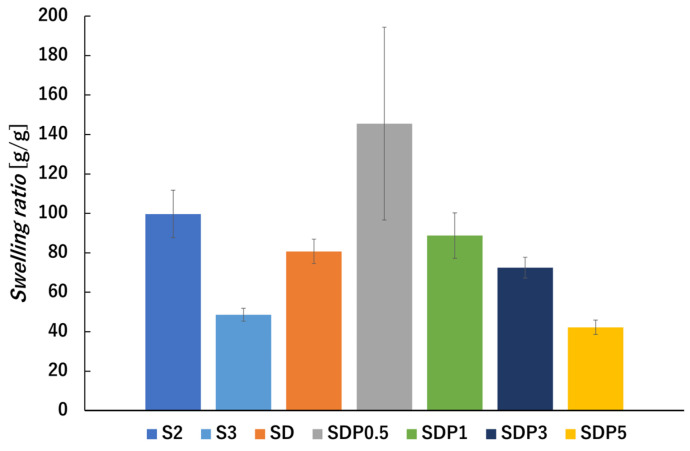
Swelling ratios of gel beads after immersion in PBS [g/g]. The swelling ratio was measured after 24 h of immersion (*p*-values are summarized in Table S3).

In the case of the swelling measurement in PBS (Figure 13), the swelling ratio of gel beads other than S2, SD, and SDP0.5 were lower than those observed in saline solution. The increased swelling of S2 and SD may be related to interactions between phosphate ions and calcium alginate, as reported previously [13]. In addition, the decrease in swelling ratio with increasing PAsp content may be associated with contraction of PAsp chain under high ionic strength conditions, consistent with the NaCl concentration-dependent swelling behavior shown in Figure 12. Taken together, the swelling results in distilled water, saline, and PBS demonstrate that PAsp does not uniformly enhance swelling, but rather appears to modify the balance between ionic screening, ion exchange, and polymer chain contraction depending on the aqueous environment.

### 3.5. Metal Adsorption Property

**Figure 14:**
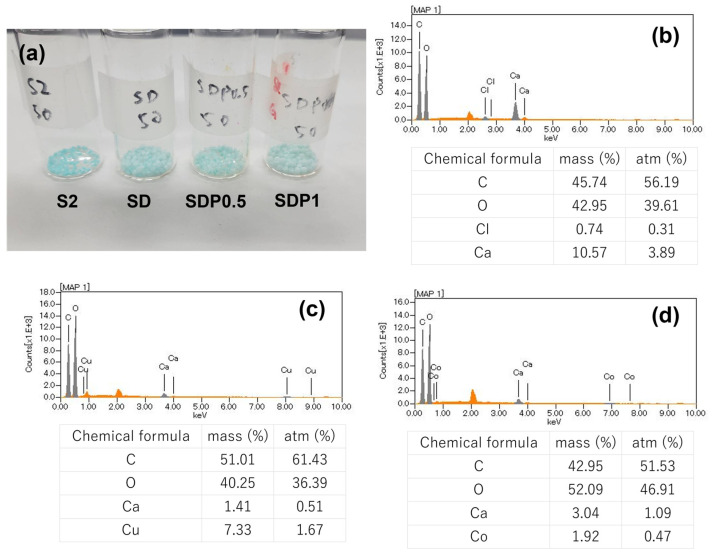
Photograph of SA/PAsp gel beads after immersion in Cu^2+^ solution (**a**) and EDS analysis of prepared SDP0.5 gel beads before adsorption (**b**) and after 24 h immersion in Cu^2+^ (**c**) and Co^2+^ (**d**) solutions.

Alginic acid and poly aspartic acid interact with various metal ions and have gelation and aggregation properties. In this study, the metal adsorption behavior of the prepared gel beads was investigated using copper and cobalt ions as representative harmful metal species commonly found in industrial wastewater. All adsorption experiments were conducted under the same conditions as described in the Section 2.7, using a fixed amount of gel beads and solution volume, with stirring at 150 rpm for 24 h. Aqueous solutions containing 50 ppm Cu^2+^ or Co^2+^ were prepared, and the gel beads were immersed to evaluate their relative metal adsorption behavior. The metal adsorption behavior of the prepared gel beads was evaluated by combination of visual observation, EDS analysis, and UV–vis spectroscopy. The ratio of metal atoms inside the gel beads before and after adsorption was analyzed by EDS analysis after lyophilized gel beads. S2, SD, SDP0.5, and SDP1 gel beads as samples were selected for this measurement. The appearance of the gel beads after metal adsorption and EDS results are shown in Figure 14 and Figures S2 and S3.

From Figure 14a, SA gel beads exhibited a light blue color. In contrast, the blue coloration of SD and SDP gel beads was lighter than that of S2. This difference may reflect variations in the internal packing state and network structure of the gel beads arising from differences in polymer composition and preparation conditions, rather than directly indicating adsorption capacity.

**Figure 15:**
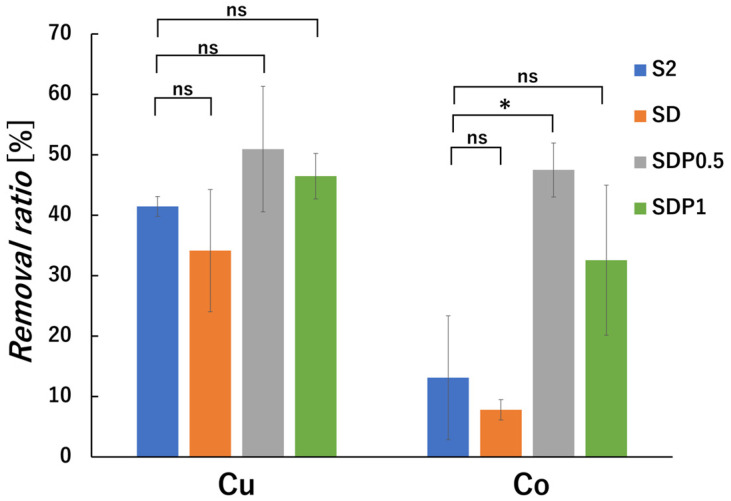
Removal ratios of Cu^2+^ and Co^2+^ ions by gel beads at an initial concentration of 50 ppm. The adsorption experiments were conducted for 24 h. Statistical significance was evaluated by Welch’s *t*-test. ns indicates no significant difference, while * indicates a statistically significant difference (*p* < 0.05).

From the results of EDS shown in Figure 14b,c, copper was detected after the adsorption experiment, suggesting the presence of copper was associated with the gel beads. The elemental ratios obtained from EDS showed that the detected copper content was higher than that of calcium. In addition, cobalt was also detected in the gel beads (Figure 14d), although the detected amount was lower than that of copper. In this study, EDS analysis was used as a supplementary method to confirm the presence of metal ions on the gel beads after adsorption, and not assess adsorption mechanisms or spatial distribution. To further verify the removal of metal ions from the aqueous phase, the metal concentrations in the solution before and after immersion were quantified by UV–vis spectroscopy using calibration curves (Figure 15).

Calibration curves for each metal ion were prepared according to the experimental method (Figures S4 and S5). From the result of Figure 15, all samples exhibited adsorption of Cu^2+^ and Co^2+^ at initial concentration of 50 ppm. The SDP0.5 gel beads showed higher metal removal ratios than S2 and SD for both Cu^2+^ and Co^2+^. The lower adsorption observed for SD compared with S2 may be related to the reduction in G-block content in the alginate chain during ADA preparation, which can suppress the formation of egg-box structures. This observation suggests that G-block-rich regions contribute to metal ion adsorption in alginate-based gel systems. The increased metal ion adsorption observed for SDP0.5 gel beads may be associated with the introduction of additional carboxylate functional groups derived from PAsp. While SA and SD gel beads exhibited relatively low Co^2+^ adsorption, PAsp-containing gel beads exhibited enhanced Co^2+^ adsorption, suggesting a contribution of PAsp to the adsorption tendency. Although no statistically significant difference was observed, the slightly lower Co^2+^ adsorption for SDP1 may be related to changes in the gel network structure at higher PAsp content. The increased crosslink density may reduce the accessibility of effective carboxylate sites and increase diffusion resistance for Co^2+^ ions. In addition, metal ion adsorption behavior is influenced by solution acidity, as metal ions and protons can compete for the same adsorption sites under highly acidic conditions [42,43]. At low pH, partial protonation of carboxylic groups in PAsp may further suppress ion exchange with metal ions [44]. Acid/base tolerance measurements (Figure S1b) showed that SD and SDP gel beads were not stable under acidic conditions; nevertheless, SDP gel beads still exhibited the ability to adsorb Cu^2+^ and Co^2+^, indicating their relative adsorption tendency under the tested conditions.

Overall, these results demonstrate the comparative influence of PAsp incorporation on metal ion adsorption behavior in alginate gel beads under identical experimental conditions.

### 3.6. Dye Adsorption Property

**Figure 16:**
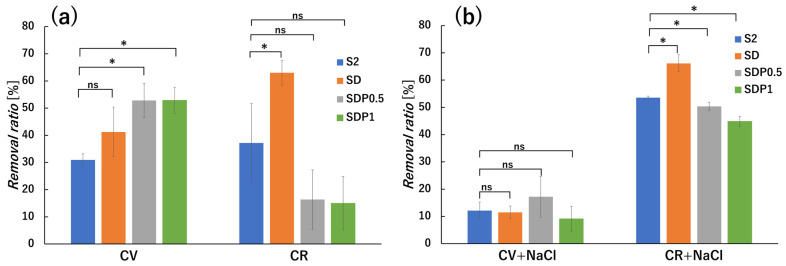
Removal ratio of dyes by gel beads in (**a**) distilled water and (**b**) saline solution. The adsorption experiments were conducted for 24 h. Statistical significance was evaluated by Welch’s *t*-test. ns indicates no significant difference, while * indicates a statistically significant difference (*p* < 0.05).

All adsorption experiments were conducted under the same conditions as described in the Section 2.8, using a fixed amount of gel beads and solution volume, with stirring at 150 rpm for 24 h. The property of dye adsorption on SA/PAsp gel beads was investigated using crystal violet (CV) as a cationic dye and Congo red (CR) as an anionic dye [45,46]. Dye solutions were prepared at 10 mg/L and a pH of approximately 7, and the concentration of the dye solutions before and after adsorption was measured by UV–vis (Figure 16a) based on calibration curves constructed from Figure S6. Adsorption experiments were conducted at a fixed initial concentration and pH to enable direct comparison of the relative effects of PAsp incorporation on dye adsorption behavior among different gel compositions.

From the results, the removal ratio of CV increased with the addition of PAsp, whereas the removal ratio of CR decreased with increasing PAsp content. This charge-dependent adsorption behavior suggests that electrostatic interactions contribute to the observed adsorption trends. Similar selective adsorption of cationic and anionic dyes depending on the surface charge of polysaccharide-based hydrogels has been reported in alginate- and chitosan-based systems [47]. The increased CV adsorption with PAsp incorporation may be associated with the higher density of carboxylic groups introduced by PAsp, which can enhance electrostatic attraction toward cationic dye molecules. According to the research of Akin et al., hydrogen bonding involving hydroxyl groups can also contribute to CV adsorption onto gel beads [48]. Therefore, the enhanced CV adsorption observed in PAsp-containing gel beads is likely governed by a combination of electrostatic interaction and secondary interaction such as hydrogen bonding, rather than by a single dominant mechanism.

In contrast, CR adsorption decreased with increasing PAsp content. Chatterjee et al. reported that CR interacts with polysaccharide-based adsorbents through physical interactions such as hydrogen bonding and van der Waals forces rather than strong electrostatic attraction [49]. In the present system, CR may preferentially interact with hydrogen bonding sites in the alginate-rich domains rather than with PAsp-rich regions. The relatively high removal ratios observed for S2 and SD samples may also be partially influenced by precipitation of CR under the experimental conditions, which can contribute to an apparent increase in removal. In addition, the higher apparent adsorption observed for SD may be related to possible interactions between amino groups of CR and aldehyde groups of ADA, leading to imine formation, as reported previously [50]. With increasing PAsp content, electrostatic repulsion between negatively charged PAsp chains and anionic CR molecules is likely enhanced, thereby suppressing CR adsorption.

Dye adsorption from saline solutions was also performed to investigate the effect of salt (Figure 16b). The concentrations of dye solutions before and after adsorption were measured by UV–vis spectroscopy using the calibration curves shown in Figure S7. From the result, the removal ratio of CV decreased markedly, whereas CR adsorption for SDP0.5 and SDP1 increased in the saline solution. The decrease in CV adsorption under saline conditions may be attributed to competition between sodium ions and cationic dye molecules for adsorption sites on the gel beads. For CR adsorption, the increased removal observed in all samples, particularly in SDP gel beads, may be associated with swelling-induced changes in the gel structure under saline conditions. Ion exchange between calcium and sodium ions can promote swelling of the gel network, which may increase the availability of functional groups such as carboxylic and hydroxyl groups that were previously involved in ionic crosslinking, thereby enhancing hydrogen bonding with dye molecules. It should be noted that this interpretation is based on macroscopic swelling behavior rather than direct spectroscopic evidence of specific binding interactions.

**Figure 17:**
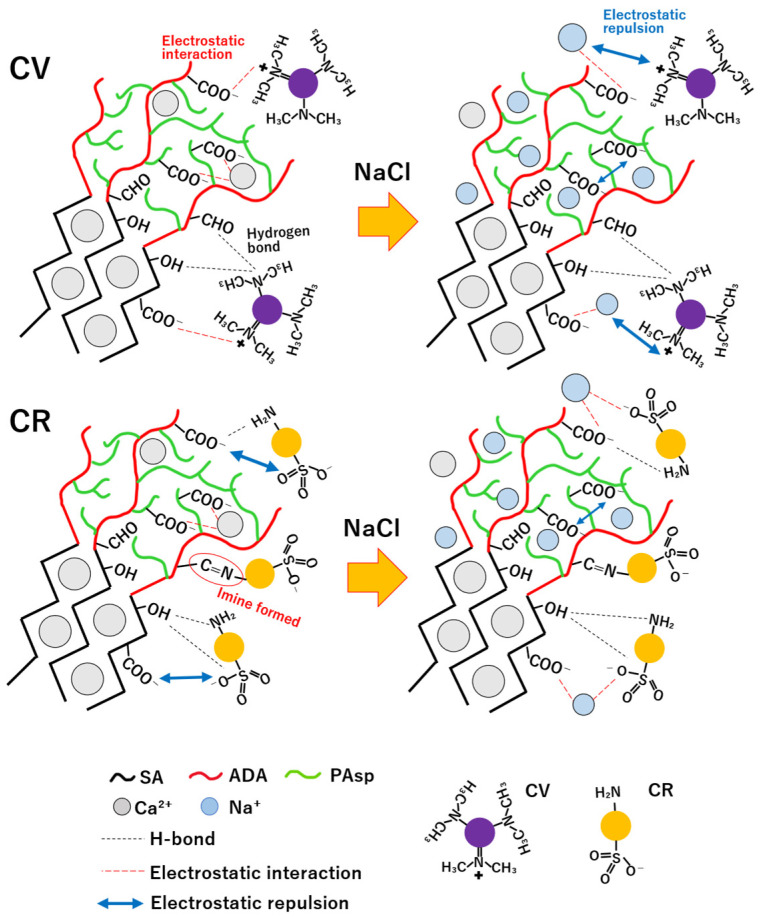
Schematic illustration of the proposed behavior of crystal violet (CV) and Congo red (CR) on SDP gel beads in distilled water and saline solution.

This interpretation is supported by recent research on alginate-based hydrogel adsorbents, which emphasize that dye adsorption can proceed through a combination of electrostatic interactions, hydrogen bonding, and structural changes in the polymer network depending on solution conditions [51]. Based on these observations, a schematic illustration summarizing the proposed adsorption behavior of CV and CR on SDP gel beads under distilled water and saline conditions is shown in Figure 17.

Overall, sodium ions are suggested to influence dye adsorption by modifying electrostatic interactions and the availability of adsorption sites on the gel beads. The removal ratio of cationic dyes decreases due to competition between cations and electrostatic repulsion, whereas the removal ratio of anionic dyes increases due to the reduced electrostatic repulsion and enhanced hydrogen bonding interactions.

These results indicate that PAsp incorporation affects dye adsorption behavior in a charge-dependent manner, and that electrostatic interactions play an important but not exclusive role in governing the observed adsorption tendencies under the tested conditions.

## 4. Conclusions

In this study, hydrogel beads composed of alginate and poly aspartate were successfully prepared via Ca^2+^ crosslinking and ADA-mediated covalent bonding. The incorporation of PAsp into the alginate network was found to influence the swelling behavior of the gel beads in distilled water, saline solutions, and phosphate-buffered saline solution, indicating that the swelling response can be modulated by polymer composition under different aqueous environments.

Metal adsorption experiments revealed that PAsp-containing gel beads exhibited relatively enhanced adsorption tendencies toward Cu^2+^ and Co^2+^ compared with alginate-only systems under identical experimental conditions, achieving metal removal ratios of approximately 40–50% at an initial concentration of 50 ppm. Dye adsorption experiments showed that PAsp incorporation affected adsorption behavior depending on the dye charge, indicating that electrostatic interactions together with functional group availability contribute to the observed adsorption tendencies.

The results clarify the relative effects of PAsp incorporation on the swelling and adsorption behavior of alginate-based hydrogel beads and provide fundamental insight into structure–property relationships of polymer-based hydrogel systems. These findings align with and provide experimental support for recent research on alginate-based hydrogel absorbents for water treatment, which highlight the importance of polymer network design and functional group incorporation in governing swelling and adsorption behavior [46]. While the present study focused on comparative trends, the results provide a basis for understanding how polymer composition influences hydrogel behavior under different aqueous conditions.

Future work will focus on systematic adsorption isotherm and kinetic analyses, adsorption–desorption cycle tests to evaluate reusability, and further quantitative evaluation of the biodegradation behavior of the prepared gel beads.

## Figures and Tables

**Table 1:** Polymer content of gel beads.

(wt%)	SA	ADA	PAsp	Total
S2	2	-	-	2
S3	3	-	-	3
SD	1	1	-	2
SDP0.5	1	0.5	0.5	2
SDP1	1	1	1	3
SDP3	1	1	3	5
SDP5	1	1	5	7

## Data Availability

The raw data supporting the conclusions of this article will be made available by the authors on request.

## Supplementary Materials

The following supporting information can be downloaded at: https://www.mdpi.com/article/10.3390/polym18020177/s1, Figure S1: Acid/base tolerance test of gel beads in the pH range of 1–11. (a) Swelling ratio [g/g] after immersion in solutions of different pH and (b) weight loss [%]; Figure S2: EDS analysis of S2, SD, SDP0.5, and SDP1 gel beads before adsorption (a–d) and after immersion in Cu^2+^ solution (a’–d’); Figure S3: EDS analysis of S2, SD, SDP0.5, and SDP1 gel beads after immersion in Co^2+^ solution (a–d); Figure S4: Calibration curve of Cu^2+^ standard solution prepared in 0.1 M nitric acid; Figure S5: Calibration curve of Co^2+^ standard solution prepared in 0.1 M nitric acid; Figure S6: Calibration curves of (a) crystal violet and (b) Congo red prepared in distilled water; Figure S7: Calibration curves of (a) crystal violet and (b) Congo red prepared in saline solution; Table S1: Pairwise *p*-values (DI water, *n* = 3); Table S2: Pairwise *p*-values (saline solution, *n* = 3); and Table S3: Pairwise *p*-values (PBS, *n* = 3) [51,52,53,54].
