# Supplementary material for: Swelling Property and Metal Adsorption of Dialdehyde Crosslinked Poly Aspartate/Alginate Gel Beads
Source: Polymers (Basel). 2026 Jan 8;18(2):177. doi: 10.3390/polym18020177 (PMC12845902; doi:10.3390/polym18020177)
Supplement: Supplementary file 1 [file polymers-18-00177-s001.zip › polymers-4051601-supplementary.pdf]

polymers

MDPI

Article

Swelling property and metal adsorption of dialdehyde crosslinked poly aspartate /alginate gel beads

Takuma Yamashita and Toshihisa Tanaka

Support information

S.1 p-value of distilled water, saline solution and PBS swelling ratios

This support information provides complete matrices of pairwise p-values obtained from Welch's two-tailed t-tests for swelling ratios measured in distilled water, saline, and PBS.

Table S1. Pairwise p-values (DI water, n = 3)

	S2	S3	SD	SDP0.5	SDP1	SDP3	SDP5
S2	-	**0.010**	**0.033**	**0.047**	**0.047**	**0.004**	**0.007**
S3		-	0.059	0.090	0.070	**0.004**	**0.007**
SD			-	0.829	0.317	**0.008**	**0.019**
SDP0.5				-	0.270	**0.010**	**0.020**
SDP1					-	0.130	0.249
SDP3						-	0.346
SDP5							-

Table S2. Pairwise p-values (Saline solution, n = 3)

	S2	S3	SD	SDP0.5	SDP1	SDP3	SDP5
S2	-	**0.032**	**0.088**	**0.002**	**< 0.001**	**0.002**	0.954
S3		-	**0.002**	**0.004**	**0.002**	**0.003**	0.057
SD			-	**0.006**	**0.002**	**0.007**	0.152
SDP0.5				-	0.104	**0.016**	**0.002**
SDP1					-	**0.034**	**0.001**
SDP3						-	**0.003**
SDP5							-

Table S3. Pairwise p-values (PBS, n = 3)

	S2	S3	SD	SDP0.5	SDP1	SDP3	SDP5
S2	-	**0.013**	0.094	0.243	0.319	**0.043**	0.065
S3		-	**0.004**	0.075	**0.020**	**0.005**	**<0.001**
SD			-	0.147	0.366	0.157	0.311
SDP0.5				-	0.177	0.121	0.131
SDP1					-	0.120	0.184
SDP3						-	0.430
SDP5							-

 https://doi.org/10.3390/polym18020177

S.2 Acid/Base tolerance

Swelling behavior at different pH (pH1-13) and acid/base dissolution tolerance of the gel beads were investigated by immersing weighed samples in aqueous solutions of different pH for 48 h. After immersion, the gel beads were isolated, washed several times with distilled water, lyophilized, and weighed. Acid/base tolerance was evaluated by comparing the weight loss ratios at different pH by values using Equation (SE1). pH adjusted solutions were prepared using concentrated hydrochloric acid and 0.1 M sodium hydroxide solution.

$$\text{Weight loss (\%)} = \frac{(W_d - W_0)}{W_0} \times 100 \qquad \text{(SE1)}$$

Where W_0 and W_d denote the initial weight and dried weight of gel beads after immersing, respectively.

Acidic and basic solutions in the pH 1.0 - 13 were prepared using concentrated hydrochloric acid and 0.1 M sodium hydroxide solution. The changes in weight and macroscopic appearance before and after immersion were examined to assess acid and base resistance (Figure S1).

From the swelling ratio results (Figure S1. (a)), the gel beads exhibited relatively low swelling ratios under acidic conditions. This behavior may be attributed to ion exchange between calcium ions and hydrogen ions, which can weaken the ionic crosslinks in calcium alginate and promote conversion toward alginic acid-rich domains. Similar pH-sensitive weakening behavior of Ca^{2+}-crosslinked alginate gels under acidic environments has been reported previously, where partial disruption of ionic interactions was observed rather than complete network collapse [52]. The relatively higher swelling observed for PAsp-containing gel beads under acidic conditions should be interpreted cautiously, as it may reflect differences in overall network looseness and hydrophilicity rather than a specific binding mechanism. Possible contributing factors include (i) variations in internal network packing, (ii) the inherently hydrophilic nature of PAsp, and (iii) partial suppression of interchain hydrogen bonding within alginate chains. However, these interpretations are based on macroscopic swelling behavior and were not directly verified by spectroscopic or structural analyses in this study.

Under strongly basic conditions, all gel beads dissolved at pH 13. This dissolution is generally associated with extensive ion exchange between calcium and sodium ions, as well as alkaline degradation of polysaccharide chains, leading to loss of network integrity. In moderately basic solutions (pH 9-11), gel beads containing ADA and PAsp exhibited higher swelling ratios compared with alginate-only gel beads. This trend may be related to a lower effective crosslink density caused by the presence of ADA and PAsp, which can facilitate polymer chain relaxation under basic conditions, rather than indicating enhanced structural stability.

Weight loss results (Figure S1. (b)) showed that all gel beads samples exhibited measurable weight loss after immersion in acidic solutions. This behavior may arise from weakening of the gel network due to protonation of carboxylate groups and subsequent partial dissolution of polymer chains. PAsp-containing gel beads exhibited relatively higher weight loss than alginate-only gel beads, particularly for SDP3 and SDP5 samples. This trend may reflect the combined effects of lower ionic crosslink density and increased hydrophilicity, as well as possible leaching of loosely bound or uncrosslinked PAsp fractions during immersion and washing steps. It should also be noted that the washing process itself may contribute to polymer loss once the gel network has been weakened by ion exchange. Overall, these results indicate that gel beads containing ADA and PAsp can swell under acidic and basic conditions, however, they exhibit reduced structural tolerance

compared with alginate-only gel beads. The observed acid/base responses are consistent with known pH-sensitive behavior of calcium-crosslinked alginate systems and should be interpreted as comparative trends rather than as evidence of specific molecular mechanisms.

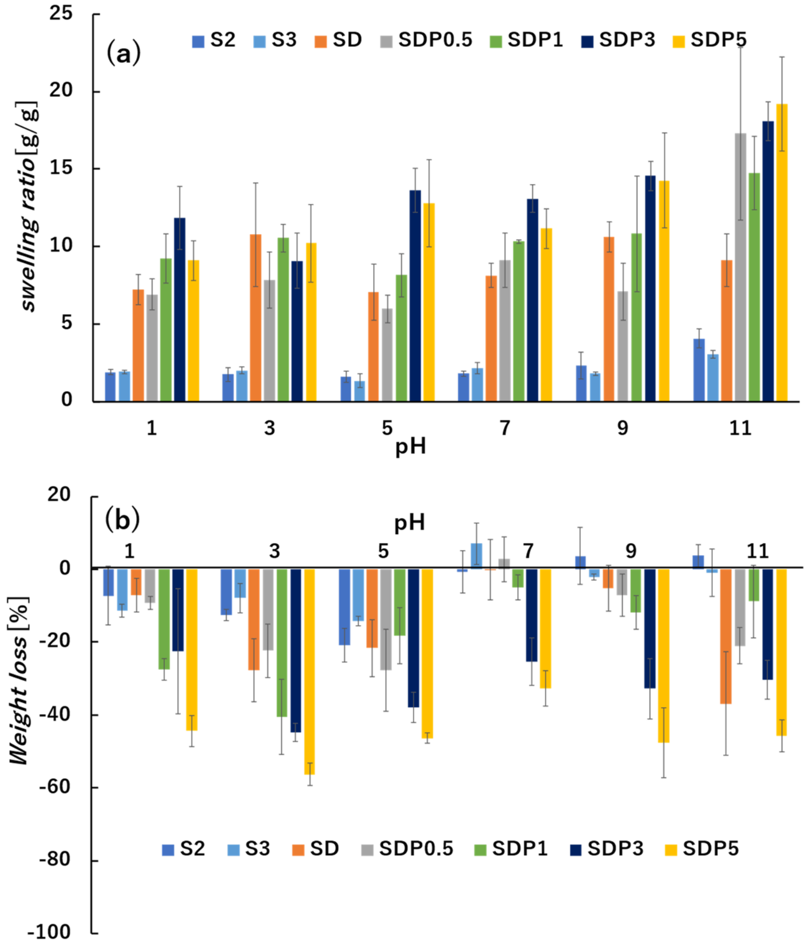

Figure S1. Acid/base tolerance test of gel beads in the pH range of 1-11. (a) Swelling ratio [g/g] after immersion in solutions of different pH and (b) weight loss [%].

S.3 EDS result of Metal adsorption

Metal ion contents in the gel beads before and after immersed in 50 ppm standard metal ion solutions, were investigated by SEM/EDS. EDS results are shown in Figures S2, S3 for copper and cobalt elements, respectively. From the result, copper and cobalt elements adsorbed in S2, SD, SDP0.5, and SDP1 gel beads after immersion.

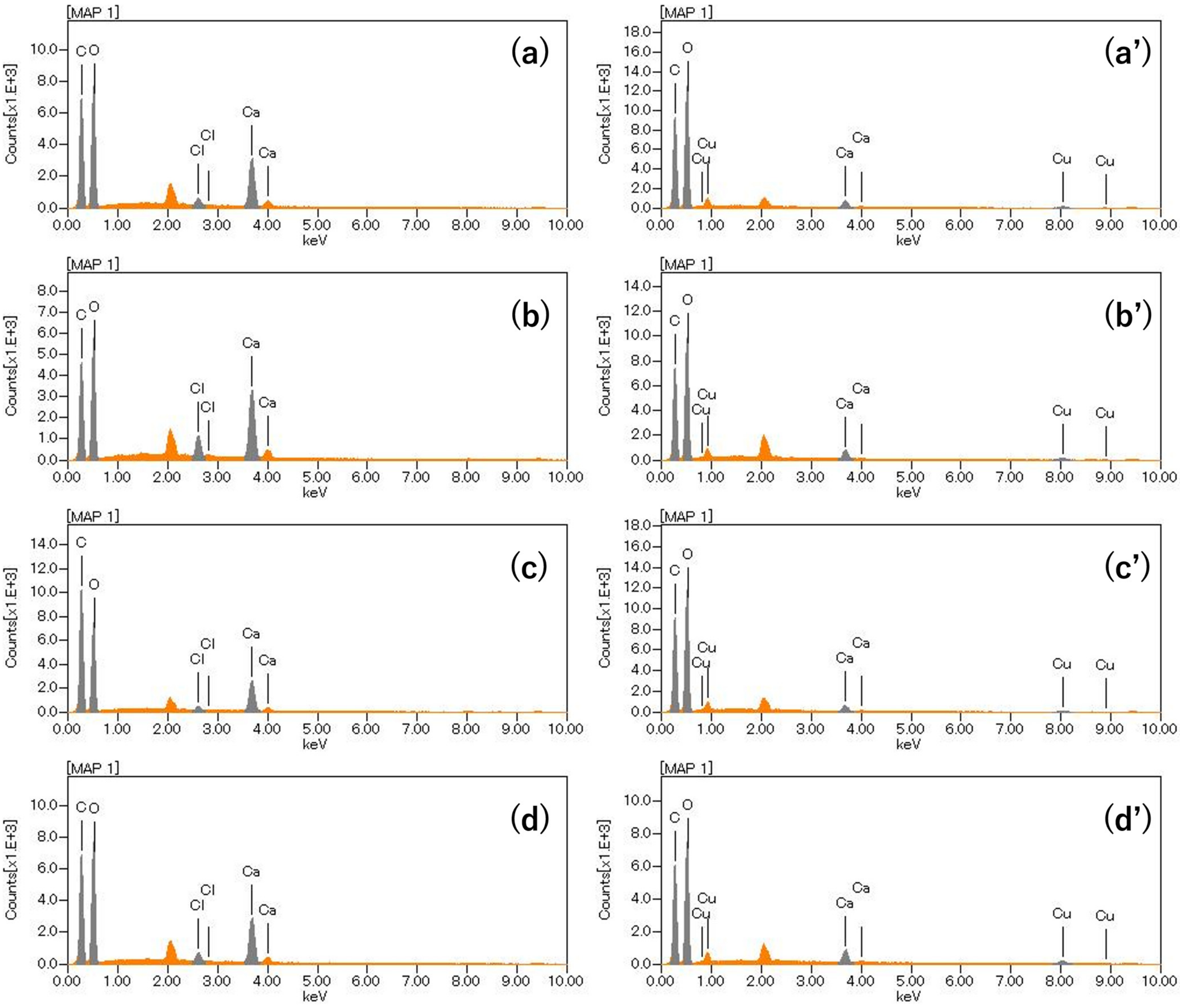

Figure S2. EDS analysis of S2, SD, SDP0.5, and SDP1 gel beads before adsorption (a)-(d) and after immersion in Cu^{2+} solution (a')-(d').

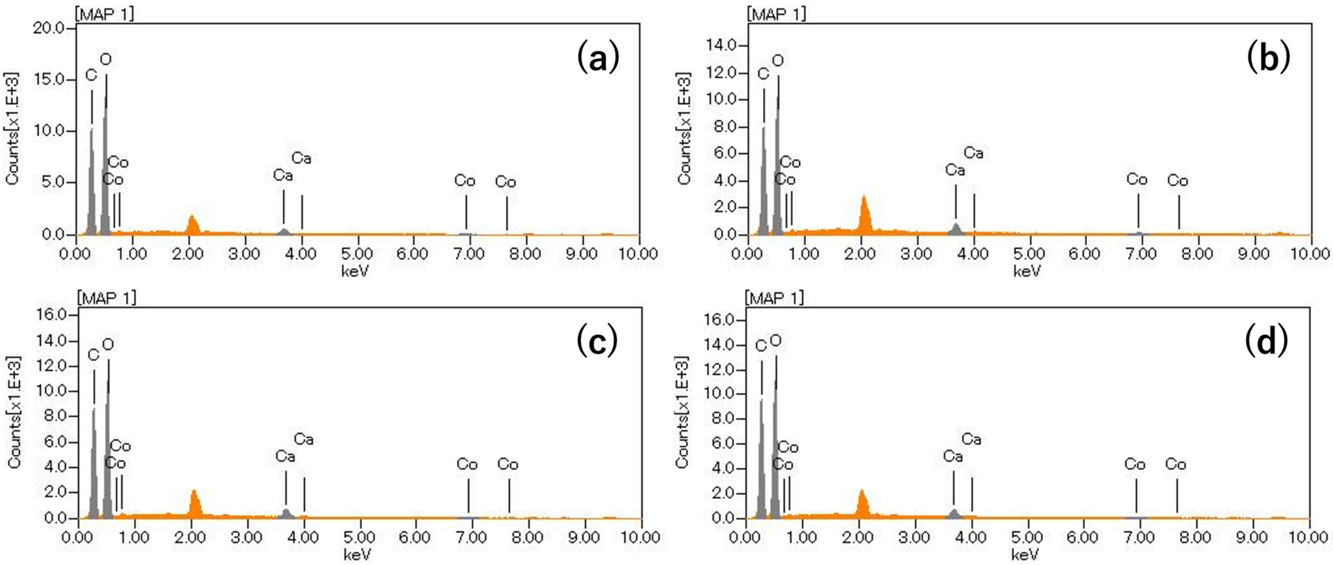

Figure S3. EDS analysis of S2, SD, SDP0.5, and SDP1 gel beads after immersion in Co^{2+} solution (a)-(d).

S.4 UV–Vis Calibration Curves for Dye and Metal Ion Quantification

Calibration curves for Cu^{2+} and Co^{2+} ions were prepared to quantify changes in metal ion concentration before and after adsorption experiments. Commercially available 1000 ppm metal ion standard solutions, in which copper nitrate and cobalt nitrate were dissolved in 0.1 M nitric acid, were used as stock solutions. These stock solutions were diluted with distilled water using volumetric flask to prepare calibration solutions with concentrations of 0, 100, 200, 500, 1000 ppm.

UV-Vis absorbance measurements were conducted at characteristic wavelengths of 806 nm for Cu^{2+} and 512 nm for Co^{2+}, based on reported absorbance features of nitrate salt solutions [53]. Calibration curves were constructed using absorbance values measured against matrix-matched blank solutions containing the same nitric acid concentration as the corresponding standard solutions. This approach ensured that background absorption from nitric acid was properly accounted for in each measurement. Although each calibration point was measured once, the calibration curves exhibited excellent linearity over the investigated concentration range, with correlation coefficients (R^2) of approximately 0.999 for both Cu^{2+} and Co^{2+} (Figures S4 and S5). These results indicate that calibration method was sufficiently reliable for evaluating relative concentration changes in metal ion solutions before and after adsorption experiments. The calibration curves were used to determine metal ion removal ratios rather than to establish absolute analytical precision. Therefore, error bars were not included for calibration data.

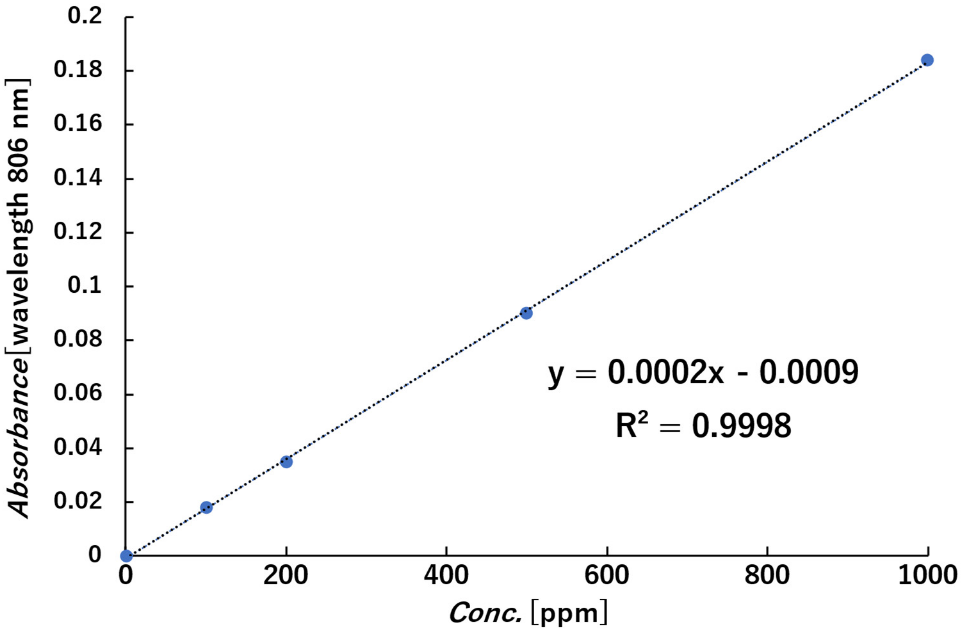

Figure S4. Calibration curve of Cu^{2+} standard solution prepared in 0.1 M nitric acid.

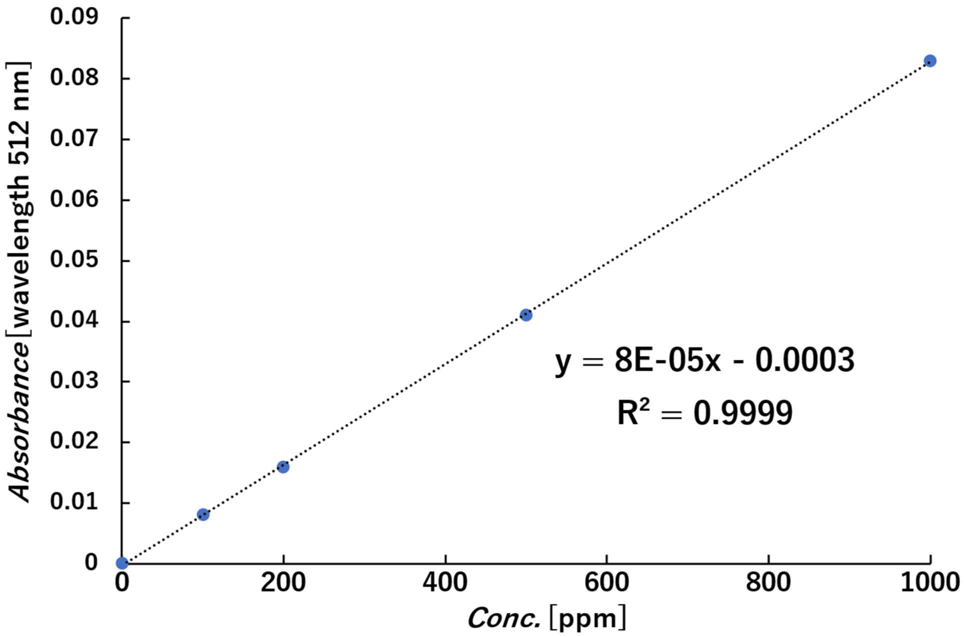

Figure S5. Calibration curve of Co^{2+} standard solution prepared in 0.1 M nitric acid.

Crystal violet and Congo red were selected as dyes, and their standard solutions were prepared at concentrations of 1-20 mg/L and 5-30 mg/L in distilled water, respectively. The absorbance of each dye at a specific wavelength was obtained by UV-Vis spectroscopy. The specific wavelengths used to construct the calibration curves for crystal violet and Congo red were 590 nm and 498 nm, respectively [51,54]. The calibration curve of crystal violet in distilled water is shown in Figure S6(a), and Congo red in distilled water is shown in Figure S6(b). In addition, calibration curves of crystal violet and Congo red in saline solution were also prepared at concentration of 1-20 mg/L and 5-30 mg/L, respectively. The specific wavelength for calibration curves of crystal violet and Congo red was 590 nm and 486 nm, respectively. The calibration curves of crystal violet and Congo red in saline are shown in Figure S7(a) and (b), respectively.

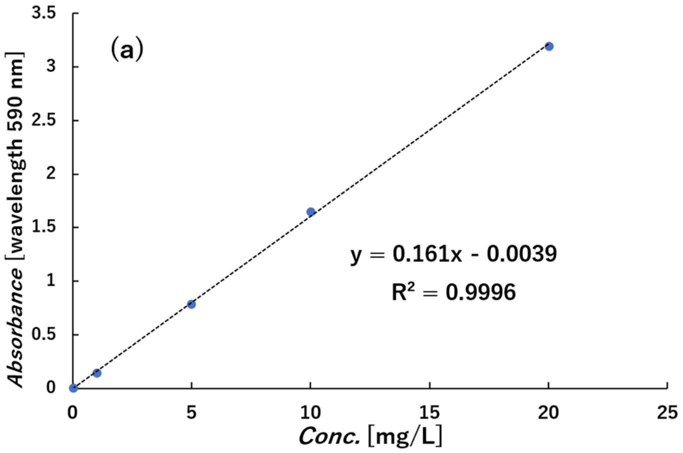

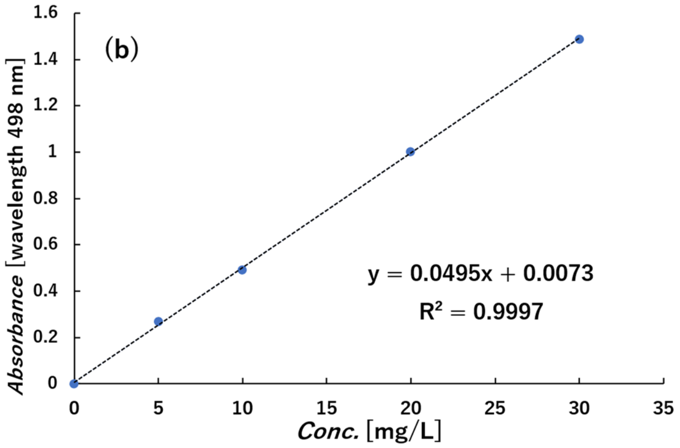

Figure S6. Calibration curves of (a) crystal violet and (b) Congo red prepared in distilled water.

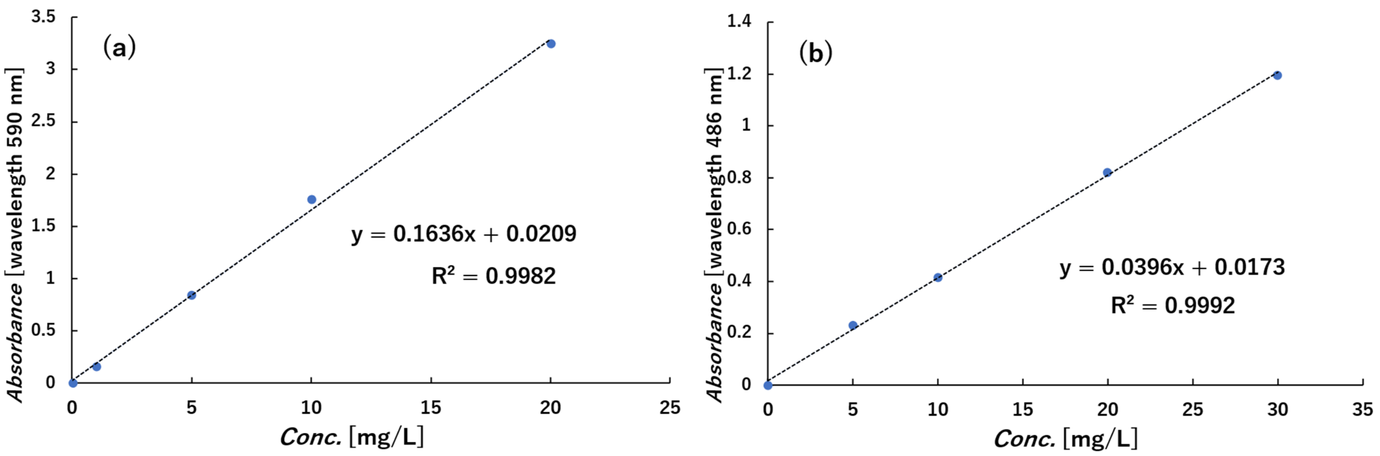

Figure S7. Calibration curves of (a) crystal violet and (b) Congo red prepared in saline solution.

Reference

51. Saket, P.; Nagpure, G.; Bala, K.; Joshi, A. Unlocking the Potential of Chitosan and Alginate for Congo Red Dye Removal by Economical Modifications in Batch and Column Study. *Biotechnol Sustain Mater* **2025**, *2*, 23, doi:10.1186/s44316-025-00043-0.
52. Malektaj, H.; Drozdov, A.D.; deClaville Christiansen, J. Swelling of Homogeneous Alginate Gels with Multi-Stimuli Sensitivity. *IJMS* **2023**, *24*, 5064, doi:10.3390/ijms24065064.
53. Liang, Q.; Jiang, L.; Zheng, J.; Duan, N. Determination of High Concentration Copper Ions Based on Ultraviolet—Visible Spectroscopy Combined with Partial Least Squares Regression Analysis. *Processes* **2024**, *12*, 1408, doi:10.3390/pr12071408.
54. Mittal, A.; Mittal, J.; Malviya, A.; Kaur, D.; Gupta, V.K. Adsorption of Hazardous Dye Crystal Violet from Wastewater by Waste Materials. *Journal of Colloid and Interface Science* **2010**, *343*, 463–473, doi:10.1016/j.jcis.2009.11.060.
